# Stigma in Pediatric Cancer: An Exploratory Study of Osteosarcoma and Retinoblastoma in Guatemala, Jordan, and Zimbabwe

**DOI:** 10.1200/GO.24.00017

**Published:** 2024-06-21

**Authors:** Dylan E. Graetz, Thelma Velasquez, Inam Chitsike, Hadeel Halalsheh, Ana Cáceres-Serrano, Lucia Fuentes, Nester Chokwenda, Edith Matsikidze, Gia Ferrara, Tharwa Bilbeisi, Anneliese Williams, Nickhill Bhakta, Sima Jeha, Carlos Rodriguez Galindo, Jennifer W. Mack, Victor M. Santana

**Affiliations:** ^1^ St Jude Children's Research Hospital, Memphis, TN; ^2^ Unidad Nacional de Oncología Pediátrica, Guatemala City, Guatemala; ^3^ University of Zimbabwe and Parirenyatwa Hospital, Harare, Zimbabwe; ^4^ King Hussein Cancer Center, Amman, Jordan; ^5^ Purdue University, West Lafayette, IN; ^6^ Dana Farber Cancer Institute/Boston Children's Hospital, Boston, MA

## Abstract

**PURPOSE:**

Stigma is an understudied barrier to health care acceptance in pediatric oncology. We sought to explore the stigma experience, including its impact on cancer treatment decision making, and identify strategies to mitigate stigma for patients with osteosarcoma and retinoblastoma in Guatemala, Jordan, and Zimbabwe.

**METHODS:**

Participants included caregivers, adolescent patients (age 12-19 years), and health care clinicians. A semistructured interview guide based on The Health Stigma and Discrimination Framework (HSDF) was adapted for use at each site. Interviews were conducted in English, Spanish, Arabic, or Shona, audio-recorded, translated, and transcribed. Thematic analysis focused on stigma practices, experiences, outcomes, drivers, mitigators, and interventions.

**RESULTS:**

We conducted 56 interviews (28 caregivers, 19 health care clinicians, nine patients; 20 in Guatemala, 21 in Jordan, 15 in Zimbabwe). Major themes were organized into categories used to adapt the HSDF to global pediatric cancer care. Themes were described similarly across all sites, ages, and diagnoses, with specific cultural nuances noted. Pediatric cancer stigma was depicted as an isolating and emotional experience beginning at diagnosis and including internalized and associative stigma. Stigma affected decision making and contributed to negative outcomes including delayed diagnosis, treatment abandonment, regret, and psychosocial fragility. Overcoming stigma led to positive outcomes including resilience, treatment adherence, pride, and advocacy. Identified stigma drivers and mitigators were linked to potential interventions.

**CONCLUSION:**

Participants describe a shared stigma experience that transcends geography, cultural context, age, and diagnosis. Stigma manifestations have the potential to impact medical decision making and affect long-term psychological outcomes. Stigma assessment tools and interventions aimed at stigma mitigation including educational initiatives and support groups specific to pediatric cancer should be the focus of future research.

## Teaser

Stigma affects patients with pediatric cancer around the world, transcending cultural differences and affecting care.

## INTRODUCTION

The WHO defines stigma as the negative association between a person or group of people who share certain characteristics and a specific disease.^1^ It is a multilevel social process that serves as a barrier to health-seeking behavior^2^ during acute outbreaks of communicable diseases^3^ and for chronic conditions.^4-7^ Among adult patients with cancer, stigma has been associated with mental distress and decreased care utilization.^8^ In noncancer pediatric populations, perceived stigmatization has been linked to bullying, poor school performance, self-harm, and suicide.^9-12^

**CONTEXT**

**Key Objective**
How does stigma affect pediatric cancer care decision making for patients and families in Guatemala, Zimbabwe, and Jordan?
**Knowledge Generated**
The manifestations of pediatric cancer stigma, including stigma experiences and practices, are largely retained across culturally and geographically diverse global settings. Stigma affects decision making and pediatric cancer care from the time of diagnosis; our findings highlight mitigating factors and potential interventions.
**Relevance**
Oncology clinicians must recognize and address pediatric cancer stigma in order to mitigate its potential impact on outcomes.

In high-income countries, pediatric cancer stigma has demonstrated psychological impacts among survivors.^13,14^ In low- and middle-income countries (LMICs), where 90% of children with cancer live,^15^ stigma affects patient-centered care and communication,^16-20^ and has been associated with care decision making including treatment abandonment.^21,22^ Nevertheless, limited research has examined stigma for global pediatric cancer populations. The impact of stigma on treatment adherence and the mechanisms for mitigating the impact of stigma in pediatric cancer are poorly defined. Additionally, previous studies addressing pediatric cancer stigma in LMICs have been conducted predominantly as single-center studies, limiting the ability to compare how culture and context affect stigma.

The purpose of this study is to use an existing framework^2^ to explore the experience of pediatric cancer stigma and its impact on medical decision making and care acceptance in three settings: Guatemala, Jordan, and Zimbabwe. These sites were chosen for their diversity and as regions where stigma has been identified as contributing to cancer care.^16,18,19^ Within these settings, we focus on pediatric patients with retinoblastoma and osteosarcoma. The frequent need for appearance-altering surgery (enucleation or limb amputation) makes stigma particularly relevant for these diagnoses, and these populations allow for an examination of parental transference of stigma (retinoblastoma) as well as the impact of stigma on adolescent patients (osteosarcoma). We examine stigma from multiple perspectives, including pediatric patients, caregivers (defined as a parent or any person who provides daily care to the child and is involved in cancer decisions), and multidisciplinary clinicians. This cross-cultural study investigates the role stigma plays in medical decision making in the hopes of identifying potential interventions to mitigate the impact of stigma on pediatric cancer care.

## METHODS

### Setting and Participants

This qualitative study was conducted in three pediatric cancer centers, Unidad Nacional de Oncologia Pediatrica (UNOP) in Guatemala, Parirenyatwa Hospital in Zimbabwe, and King Hussein Cancer Center (KHCC) in Jordan. Guatemala is an upper-middle–income country^23^ in Central America, with a collectivist culture^24^ and a predominantly Christian and Mayan population including 24 ethnic communities. Jordan is an upper-middle–income country,^23^ with a culture heavily influenced by Arab-Islamic heritage,^25,26^ and a large refugee population. Zimbabwe, a low-income country^23^ in sub-Saharan Africa, has a predominantly Christian Bantu population,^27^ and with 16 official languages is home to many cultures and traditions. UNOP is a pediatric cancer center that treats about 500 new patients with cancer a year, Parirenyatwa is a university teaching hospital with an average of 250 new patients per year, and KHCC is a specialized cancer center with about 350-400 new pediatric cancer diagnoses a year. Patients treated at these centers have varied access to prosthetic devices and rehabilitation services. In Jordan, insurance covers most services. In Guatemala, services are provided by nongovernmental organizations. In Zimbabwe, patients must cover prostheses and outpatient rehabilitation out-of-pocket.

Three participant populations were recruited: caregivers of children with recently diagnosed osteosarcoma or retinoblastoma, newly diagnosed adolescent patients (age 12-19 years), and multidisciplinary clinicians providing care for these patients and families. Patients and caregivers were interviewed within 12 weeks of diagnosis to improve their ability to describe the impact of stigma on upfront cancer care decision making and minimize recall bias. We used purposive sampling to recruit participants. At each center, study personnel reviewed records of newly diagnosed patients to assess for eligibility. Treatment teams were notified before engagement with families. Potential participants were approached for participation at the time of scheduled clinical visits. We estimated our sample size of approximately 9-17 participants at each site and within each group (patient, caregiver, and clinician) on the basis of recent literature^28^ and conducted interviews until thematic saturation.^29^

### Study Design

Semistructured interviews were designed around a modified version of the Health Stigma and Discrimination Framework^2^ (HSDF; Data Supplement), a previously published model designed to facilitate global stigma research. Interviews focused on stigma experiences and practices as well as drivers and mitigators. The interview guide was developed in English, translated to Spanish, Shona, and Arabic, and pilot tested with 12 parents, seven patients, and four clinicians across all sites. Iterative revisions were made throughout pilot testing to accommodate linguistic and cultural considerations. For example, stigma alternatives including shame, marking, dishonor, and discrimination were included to improve comprehension. Final interview guides (Data Supplement) were back-translated into English to ensure consistency and preservation of original question intent.

### Data Collection and Analysis

Interviews were conducted in Spanish, Shona, Arabic, and English by bilingual qualitatively trained members of the research team who had no previous relationship with participants (A.C.-S., L.F., and N.C.). Interviews lasted 30-60 minutes and were audio-recorded. Recordings were transcribed and translated into English using a professional service with quality assurance by the study team.

Analysis included a combination of deductive and inductive codes. This enabled an approach that was both grounded in the literature and allowed for novel insight on the basis of rich and diverse data. A priori codes were based on the HSDF^2^ with inductive codes added through memoing and open coding of 12 transcripts. The final codebook with definitions is included in the Data Supplement. Three researchers applied finalized codes to all transcripts. Each transcript was independently coded by two members of the team; discrepancies were resolved through consensus with a third coder adjudicator. Content analysis included a framework approach on the basis of the HSDF with integration of novel themes through constant comparative analysis.^30^ Thematic analysis^31^ focused on stigma experiences and practices, impacts of stigma, stigma drivers, mitigators, and potential interventions. As themes were defined and analyzed, results were reviewed with the larger research team including global partners via web-based conferencing. MAXQDA software program (Verbi, Berlin) was used to manage data. Figures were developed using a reflexive and iterative approach. Two authors (D.E.G. and A.W.) conceptualized and drafted initial figures using Canva with constant review and revision throughout analysis.

### Confidentiality and Human Subject Protection

This study was approved by the institutional review board at St Jude Children's Research Hospital and boards responsible for human subject protection at each study site. Study processes were explained verbally in each potential participant's native language and signed informed consent was obtained.

## RESULTS

Fifty-six interviews were conducted across all sites including nine adolescents, 28 caregivers, and 19 multidisciplinary clinicians. Table 1 includes participant demographic information.

**TABLE 1:** Demographics

Variable	Guatemala	Jordan	Zimbabwe	Total
Patient				
Age, years, median (IQR)	13.5 (12-15)	15.75 (15-16.67)	14.5 (12-17)	15 (13-16.67)
Diagnosis, No.	2	5	2	9
Retinoblastoma	—	—	1	1
Osteosarcoma	2	5	1	8
Sex, No.	2	5	2	9
Male	2	3	1	6
Female	0	2	1	3
Extent of disease, No.	2	5	2	9
Localized	2	4	1	7
Metastatic	—	1	1	2
Treatment goal, No.	2	5	2	9
Curative	2	5	1	7
Palliation	—	—	1	1
Caregivers				
Age, years, median (IQR)	35.5 (30-38)	40 (28-46)	39.5 (34.5-45)	37.5 (29.5-45.5)
Child's diagnosis, No.	10	10	8	28
Retinoblastoma	5	5	5	15
Osteosarcoma	5	5	3	13
Child's sex, No.	10	10	8	28
Male	5	6	5	17
Female	5	4	3	11
Relationship, No.	10	10	8	28
Mother	9	7	7	23
Father	1	3	—	4
Grandparent	—	—	1	1
Religion, No.	11	10	8	
Islam	—	10	—	
Christian	8	—	6	
Catholic	1	—	2	
None	2	—	—	
Native language, No.	12	10	8	30
Arabic	—	10	—	10
Shona	—	—	8	8
Spanish	9	—	—	9
Indigenous language	3	—	—	3
Clinician, No.				
Occupation	8	6	5	19
Surgeon	1	1	1	3
Oncologist	1	3	—	4
Heme/onc fellow	1	—	—	1
Pediatrician	1	—	1	2
Ophthalmologist	1	—	1	2
Nurse	2	2	1	5
Social worker	1	—	—	1
Radiologist	—	—	1	1
Years of experience	8	6	5	19
1-9 years	3	4	4	11
10-19 years	3	1	—	4
≥20 years	2	1	1	4

Abbreviation: Heme/onc, hematology/oncology.

**FIG 1:**
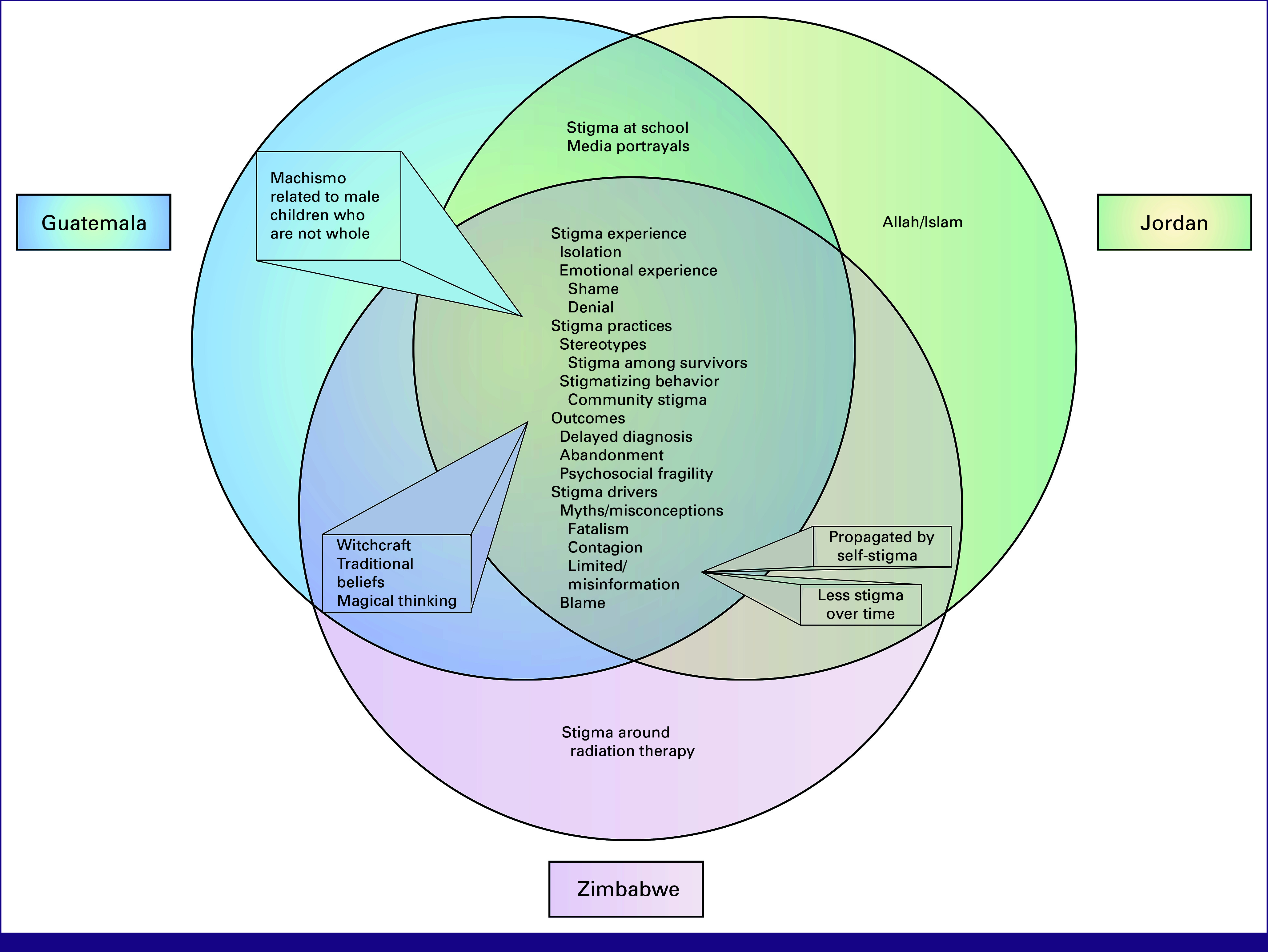
Thematic divergence and convergence. Venn diagram demonstrating thematic divergence and convergence among sites.

**FIG 2:**
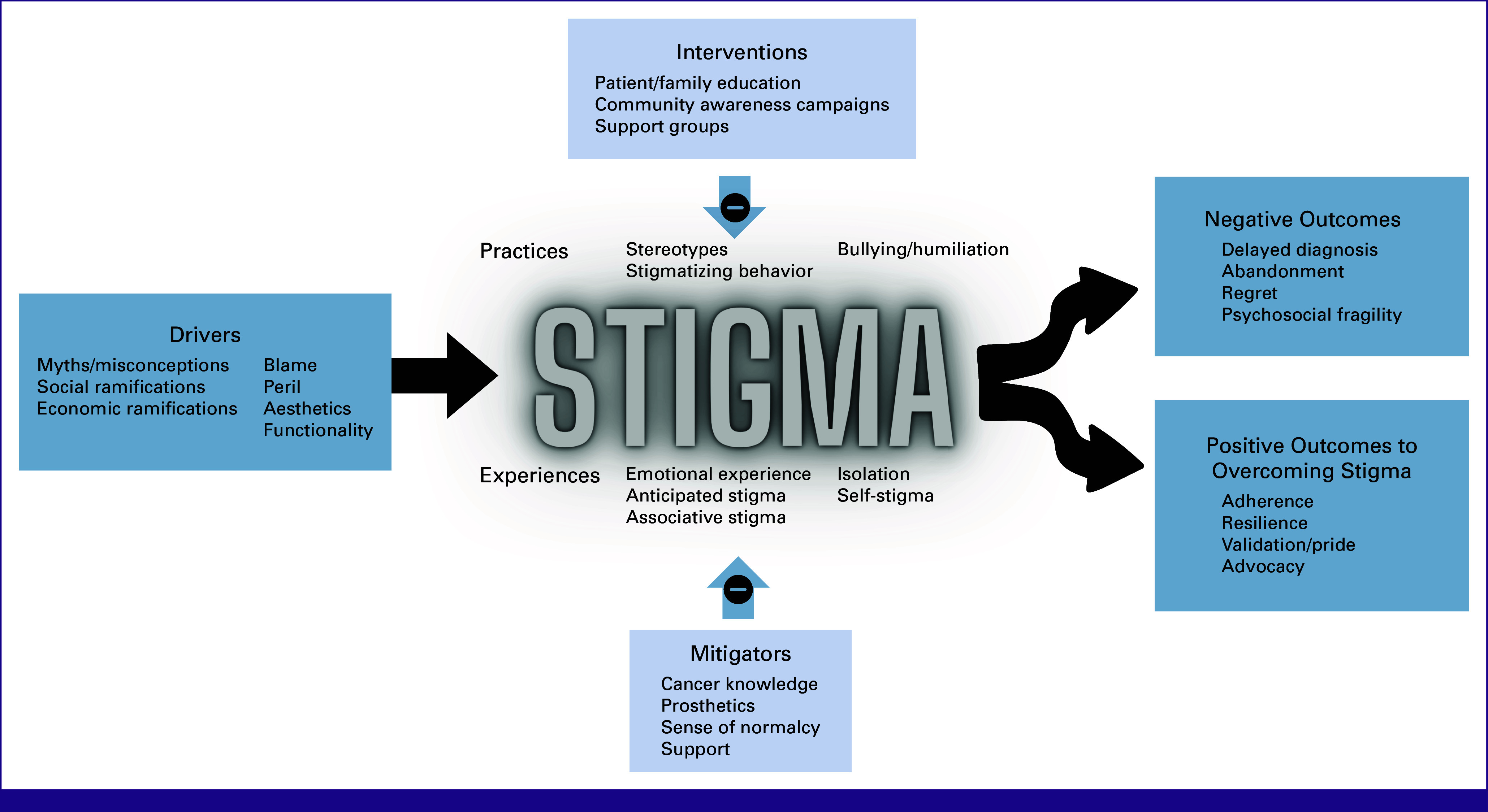
Stigma model for pediatric cancer. Stigma model adapted from the Health Stigma and Discrimination Framework as applied to global pediatric cancer.

Major themes were organized into broad categories used to adapt the HSDF^2^ to global pediatric cancer care. Overall, themes were described similarly by participants at all sites and across disease types. Occasionally, a concept was identified at only two sites. Figure 1 depicts the divergence and convergence of themes between sites. Figure 2 depicts a model for stigma generated from the data and adapted from the HSDF.^2^ Major adaptations to the model include the replacement of facilitators with mitigators and interventions, and the separation of negative outcomes and positive outcomes to overcoming stigma.

### Stigma Manifestations: Experiences and Practices

The HSDF describes stigma manifestations including stigma experiences, or lived realities of patients and families, as well as stigma practices, or beliefs, attitudes, and actions. Major themes related to stigma experiences included isolation, the emotional lived reality of stigma, anticipated stigma, internalized stigma, and associative stigma. Participants described cancer as isolating; patients and families removed themselves from the community. It was also emotional, leading to sadness, depression, fear, and exhaustion. One patient described the interplay between the emotional and isolating experience:

“Being isolated hurts the mental state of the person, it exhausts them…Some people make you lose hope, they drag your spirit down.” (Patient01, Jordan)

Anticipated stigma included concerns about future stigma experiences, started at diagnosis, and affected decision making. Caregivers and clinicians expressed concern that patients internalized stigma:

“So sometimes they stigmatize themselves, then the community will stigmatize them as well.” (Clinician01, Zimbabwe)

However, patients instead portrayed themselves as confident and self-assured:

“I am not ashamed because they took my foot away. They took it away because if they didn’t take away my foot…I could die.” (Patient02, Guatemala)

Associative stigma was particularly felt by mothers who were judged by neighbors and even abandoned by their husbands after a child’s cancer diagnosis.

Major themes related to stigma practices included stereotypes, stigmatizing behavior, and bullying/humiliation. These primarily occurred during interactions within the community or at school. Stereotypes frequently stemmed from misconceptions surrounding cancer. Stigmatizing behavior included expressions of pity or exclusion. Some participants described explicit bullying/humiliation, while others felt implicit discrimination:

“They whisper when they see me go by, or they stare at him, as its noticeable that one eye is bigger than the other, so that’s basically what people whisper about.” (Caregiver01, Guatemala)

Stigma manifestations were discussed by participants in all roles across all sites (Table 2); however, there were also patients and caregivers who denied facing stigma at all three centers.

**TABLE 2:** Stigma Experiences, Practices, and Impacts

Stigma	Participant Quotation
Stigma experience	
Isolation	“Often these children don't leave home, they are kept hidden from the community, from the curious glances of others.” (Clinician03, Guatemala)
Emotional experience	“It scares me to go out on the street with my prosthesis**…**It scares me to go out in public like that, I would want to hide it…I feel that maybe they are going to humiliate me and all that.” (Patient04, Guatemala)
Anticipated stigma	“Of course, I fear that he might be with his friends someday and they would not allow him to play because of his sickness. Of course, I have fears regarding that subject.” (Caregiver06, Jordan) “I want the best for my child and it's not easy accepting amputation. I worry about whether he will still have friends because he would be the odd one out and they may mock him or give him nicknames because of having just one leg.” (Caregiver07, Zimbabwe)
Internalized stigma	“Emotionally, emotionally, it affects the way you look at yourself you might lose your self-confidence.” (Clinician04, Zimbabwe)
Associative stigma	“Such a thing will affect both the child and the mother, she would feel that her son is shameful.” (Caregiver06, Jordan)
Stigma practice	
Stereotypes	“They had the stereotype that any patient have cancer, he will die.” (Clinician05, Jordan)
Stigmatizing behavior	“Sometimes it worries me, that someone will reject me, or humiliate me or discriminate against me or something else, yes, sometimes it worries me a lot.” (Patient04, Guatemala)
Bullying/humiliation	“Imagine when you go to school, somebody laughs at you, that person will never want to go back to school. It really affects [them].” (Clinician06, Zimbabwe)
Negative perceived impacts of stigma	
Delayed diagnosis	“When we see a patient from a very isolated rural community with little contact, we see that, even in some cases before coming here they go to the shaman, so there is a lot of mysticism, and regarding people with some level of education the approach is less complicated.” (Clinician07, Guatemala) “We've seen some cases that it was hidden by the patient and he did not contact anyone because of some stigmatization stigma and the patient referred to us in the late diagnosis.” (Clinician02, Jordan)
Abandonment	“A parent would avoid bringing their kids to get treated rather [than] face stigma and prejudice from their community of something they could get help with.” (Caregiver05, Zimbabwe) “Yeah, absolutely we do find some cases that the patient and family are refusing to do the surgery despite the, you know, despite the importance of such doing surgery. We do explain to them in detail about the possible cons. But sometimes, we do understand that some types of surgeries and we see the refusal or the rejection, especially when we are trying to do radical surgery like amputation.” (Clinician02, Jordan)
Regret	“I regret wasting time and giving my grand-son unknown portions prescribed by the traditional healer.” (Caregiver08, Zimbabwe) “And I think this question will stay in their mind forever. It never disappear. Whatever you tell them, they will keep thinking, did I do the correct decision?” (Clinician08, Jordan)
Psychosocial fragility	“It affects them massively causing their mental health to degrade, they change from what they are used to be.” (Patient05, Jordan) “We have seen many cases of children facing emotional crisis because they can't take it anymore, why continue living, why come here, because they are rejected.” (Clinician09, Guatemala)
Positive perceived impacts of overcoming stigma	
Resilience	“I feel that I will be the same as before, just with a different limb. After the surgery and all that I have felt well, I haven't felt bad, sad, I have always been happy, always trusting the doctors, who tell me that I will recover soon.” (Patient04, Guatemala) “I should never give up or compare my child to other children. We should be proud of our journey, it had already happened we cannot go back.” (Caregiver06, Jordan)
Adherence	“The thing that encouraged us is the availability of treatment. Taking treatment means the end of pain and suffering.” (Patient06, Jordan)
Validation/pride	“Thank God, I brought him to the hospital this early…I am determined to help other people recognize the importance of seeking professional medical help. I appreciate the education I received here and that is why I continue returning to this Hospital.” (Caregiver08, Zimbabwe)
Advocacy	“We seem to be making an impact slowly. But these ones that we meet with will actually [go] forward [and] become champions for us.” (Clinician10, Zimbabwe)

### Impacts of Stigma

Stigma impacts were categorized into negative outcomes consisting of four major themes: delayed diagnosis, abandonment, regret, and psychosocial fragility, and positive outcomes of overcoming stigma including resilience, adherence, validation/pride, and advocacy (Table 2).

Participants described how stigmatizing myths delayed diagnosis as caregivers sought out traditional healers. One parent explained abandonment saying:

“The negative factors do affect patients in their acceptance of healing and refusing treatment. Some would even develop despair and sadness, and they would refuse treatment completely.” (Caregiver02, Jordan)

Regret was described particularly by families or caregivers, while patients endorsed psychosocial fragility contributing to their long-term mental health and affecting outcomes:

“If they treat me differently and it shows to other people, this will affect my mental state thus affecting my results.” (Patient03, Jordan)

However, many patients and caregivers described resilience (Table 2) and validation, or strength in doing what was right. Adherence was encouraged through hope for cure and treatment availability, both of which helped families overcome stigma:

“Whatever people say, either good or bad, I have to accept it and do what's best for my child.” (Caregiver03, Zimbabwe)

Some participants described advocacy, including the desire to help others in similar circumstances and increase cancer awareness.

### Stigma Drivers

Stigma drivers are inherently negative factors that propagate stigma manifestations^2^ and downstream impacts. Major themes categorized as drivers included myths/misconceptions, blame, peril, aesthetics, functionality, social ramifications, and economic ramifications. Major themes and select subthemes are presented as column 1 of Table 3 with associated mitigators and interventions in columns 2 and 3, respectively.

**TABLE 3:** Drivers, Mitigators of Stigma, and Potential Interventions

Driver	Mitigator	Intervention
Myths/misconceptions Association with witchcraft “Most of the people thinks cancer is associated with witchcraft… So that also drives stigma.” (Clinician01, Zimbabwe) Concerns about contagion “They say that cancer is contagious and they stay away from him. Sometimes I take [my son] out to eat because some people have invited me but they say stay away because this is contagious.” (Caregiver09, Guatemala) “They would treat it as if it was a contagious disease that would contaminate them, which is not.” (Caregiver10, Jordan) That there is no treatment “Cancer has always been a sickness with a bad reputation because it did not have any treatment.” (Caregiver11, Jordan)	Cancer knowledge “These non-communicable diseases, people still need to be taught to learn a lot about them that there's nothing personal or there's no witchcraft associated with someone having a cancer or malignance.” (Clinician01, Zimbabwe) “Here they told me that it’s normal, because we don't know where it comes from or why, it's not the fault of the parents or something, this illness happens and we don't know why it affects the children, that's what they told me.” (Caregiver12, Guatemala)	Parent/family education “So perhaps using examples, contexts, showing them, explaining to them. This is an ongoing educational plan, because it's does not happen in only one session, every time they come and we see that they are worried we have to explain to them, with examples…. I believe that having a good educational plan is key.” (Clinician07, Guatemala) “So we teach the people as much as we can. Here, yeah, inside the hospital we give enough information for the families as much as we can.” (Clinician13, Jordan) “We empower them by giving them information on what to expect,” (Clinician10, Zimbabwe) “Through sitting with doctors, I have evaluated the changes that will happen to her like the hair fall and such things, let's say educating the patient and his family helps enormously.” (Caregiver02, Jordan)
Blame “They may say that it’s the parents' fault, the people in the community think that it’s something the parents did. And because of these beliefs they marginalize us.” (Caregiver12, Guatemala)		
Peril Death “100% of patients associate cancer with death.” (Clinician11, Guatemala) “At home we have this belief that there's no cure for cancer.” (Caregiver08, Zimbabwe) Loss of limb “The stress of losing a limb becomes unbearable sometimes to some of these patients.” (Clinician12, Zimbabwe) “I was very sad that they were going to amputate my leg…I felt sad and nervous and after the amputation. I still felt sad because I had lost a part, a limb of my body.” (Patient04, Guatemala)		
Aesthetics “The aesthetic aspect is also important for the patients, mostly teenagers, because they don't know how others are going to look at them, so that's a big source of anxiety, and the other thing, children with retinoblastoma after enucleation have asymmetric facial features and the parents are always trying to conceal this, they put hats on them or they cover them with a sheet. Teenagers also often try to conceal this, for example in an arm amputation they use long-sleeved shirts, the same with the leg, they try to use a sheet or a towel to hide the physical defect l, that leaves a very deep impression on them.” (Clinician03, Guatemala)	Postsurgical options “We explain that afterward we will put a prosthesis to improve the aesthetics from that point of view, so yes, we try to support them so that they accept the treatment.” (Clinician14, Guatemala) “If my son loses a part of his body, I would replace it with a synthetic one just so that people do not talk. I would replacehis removed eye with a synthetic one no matter its price because I want him to be happy and because I don't want people to talk about him.” (Caregiver13, Jordan) Sense of normalcy “That's where we [tell] them, that there are prostheses, that the eye will not be normal but that the physical appearance, showing them other children that go to school, that they can be accepted in their communities, and these people then start to think differently.” (Clinician15, Guatemala) “If you cannot tell that someone is a cancer patient, after he starts getting treatment, you will make a difference.” (Caregiver13, Jordan) “I can always play with my brother and with them. And if it is because they are going to give me a foot to walk, and it is always as if you have your foot, and you can always ride your bike, run and stuff.” (Patient02, Guatemala)	Community awareness campaigns “We should have more awareness; be more awareness about cancer types, about the survival rate, about the treatment options that we have here in KHCC or even in the worldwide.” (Clinician05, Jordan) “I think an awareness campaign to educate people who stigmatize against cancer patients better understand the realities of retinoblastoma will help curb the stigma.” (Caregiver14, Zimbabwe) “[They should] give talks about the origin of [cancer] and distribute flyers explaining that the disease is nobody's fault, it just happens.” (Caregiver12, Guatemala)
Functionality “A child who has cancer and has been through chemotherapy cannot play with those at his age, his body would be weaker.” (Caregiver11, Jordan) “For those with osteosarcoma, sometimes they will get their limb taken off, so they have to be in the wheelchair bound for the rest of their life or they have to be using some form of prosthesis to help them move around. So that also contributes [to stigma].” (Clinician01, Zimbabwe)		
Social ramifications “A lot of people refuse to marry into families of people with cancer, they fear hereditary problems, and we can face this issue.” (Caregiver04, Jordan) “They also worry about whether they'll get a job in the future. Whether they'll get a spouse, in the future, because you know with a disability.” (Clinician04, Zimbabwe)	Support Support from family “I tell my mom, and she says that no one is going to humiliate me. She says to stay calm, that in the future I will have a good job.” (Patient04, Guatemala) “We need to talk to the child and make him feel better, we need to tell him that it is a temporary situation where he will get better soon.” (Caregiver11, Jordan) “The main goal should be to help the child get treatment. Whatever people say, either good or bad, I have to accept it and do what's best for my child.” (Caregiver03, Zimbabwe) Support from the community “Now the limb is amputated and she is undergoing a process, there is an indigenous organization that is supporting them and preparing her for reinsertion in the community. They are even looking for a prosthesis so she can lead a normal life.” (Clinician09, Guatemala) “My friends came to support me, even though I was unable to go out, they came after each chemotherapy session.” (Patient01, Jordan) Support from the medical team “In a certain way, we will not fill the void in them, but in some cases we do, want we want to motivate them, to make them feel well, to prepare them and give them the tools to cope with everything they are experiencing. We tell them, today is a cloudy day, but the sun will come out tomorrow, maybe even a rainbow.” (Clinician09, Guatemala) “When I go into surgery, I don't think about it, worry or cry about it. I walk into the surgery room smiling and laughing with the doctors. One might think that I am walking into a party, I don't cry, I smile and laugh.” (Patient03, Jordan) Support from survivors “I have seen other patients receive the right medical treatment and get better and that is what gives me the strength to return to the hospital.” (Caregiver08, Zimbabwe) “I believe that when you meet someone who is living the same experience, you will benefit from them, and they will give you strength.” (Caregiver10, Jordan) “And sometimes I dare ask them how they are doing, how is their treatment going or if they finished the treatment and are here for an appointment, how did the treatment go, how did it feel, or how did they cope with so much, and they reply that always they see the doctors, the nurses and they trust the hospital.” (Patient04, Guatemala)	Support groups “Maybe a playgroup it's good for them, playgroup for patients with retinoblastoma to feel that and similar to him.” (Clinician05, Jordan) “We have a group a WhatsApp group where all cancer patients that we see can join. So in that group, there is a nurse, a representative and someone from the public relations office…they discuss all their concerns about cancer, and then the nurse representative at least if there's some form of confusion or whatever, she can always put order in to address some of the issues to ask doctors. But this is something that has just been recently introduced.” (Clinician04, Zimbabwe) “One of the mothers was saying that they identify the people that come in during the week as a group, and also depending on the diagnosis, they create a support network among them, mothers and patients, and they support each other a lot. That has been useful, on the one hand, for them to have the tools to face this and live their lives coping with these difficulties in a positive way.” (Clinician09, Guatemala) “Dealing with a sick child is never easy, so we could benefit from each other. It would make you feel better when you meet someone who has the same cause as your child.” (Caregiver04, Jordan)
Economic ramifications “In the case of parents with children with retinoblastoma, a lot of times the diagnosis affects both eyes and both have to be [enucleated], so the functional blindness affecting these patients is what scares the parents, how they are going to work in the future… they believe that knowing their disability [no one] will want to hire them in the future; that in the case of women they will not be able to do house work in the case of osteosarcoma, that they won't be able to take care of children, or become pregnant, for example. And regarding the boys, that they will not be able to work in the fields, which is what most of them usually do, planting, harvesting or working with animals, in both cases, either retinoblastoma or osteosarcoma.” (Clinician03, Guatemala)		

Stigma drivers varied more across sites than manifestations or impacts. In Zimbabwe and Guatemala, participants referenced witchcraft and traditional beliefs contributing to myths/misconceptions; these ideas were absent from Jordanian transcripts. Participants across sites described blame including how parents were blamed, or blamed themselves, for their child's cancer, and peril, the threat of cancer or losing a body part, as contributing to stigma. Aesthetic concerns, which were particularly relevant for female and adolescent patients, included specific aesthetics related to enucleation or amputation and general physical stigmata such as hair loss. One parent said:

“Other sicknesses might be internal, but cancer is reflected in the outer body, and chemotherapy can cause hair fall. Things that appear on the outside can help increase [stigma].” (Caregiver04, Jordan)

Participants also described how disability or the loss of functionality propagated stigma. Finally, participants discussed social and economic ramifications of cancer contributing to stigma, including a child's potential for marriage and ability to secure future employment.

### Stigma Mitigators and Potential Interventions

Mitigators included factors that had the potential to reduce stigma. Most major themes categorized as mitigators operated at the level of the patient or family, including availability of prosthetics, sense of normalcy, and support. However, cancer knowledge was described as mitigating stigma at the level of the individual and the community. Related to mitigators were interventions that might alleviate stigma and its impact on cancer care decision making. These included parent/family education, community awareness campaigns, and support groups. Mitigators and interventions were closely related to stigma drivers as outlined in Table 3. For example, uncertainty surrounding origin contributed to cancer myths, and limited cancer knowledge led to community misconceptions:

“I believe [it] will help [when] many have adequate knowledge of cancer. Cancer is very difficult to accept. I think it's more difficult to deal with cancer than HIV. Nobody understands where cancer comes from or what stages there are to it and what to expect and whether or not it's contagious.” (Caregiver05, Zimbabwe)

Interventions to combat disinformation included family education materials at the level of the individual, and community awareness campaigns at the community level. As one clinician said:

“Many false knowledge has been widespread all over the world. It’s important to spread the real knowledge and the important knowledge.” (Clinician02, Jordan)

Participants discussed how postsurgical options, such as prosthetics and rehabilitation services, mitigated stigma and increased care acceptance by restoring a sense of normalcy. Finally, participants highlighted psychological support from family, the community, the medical team, other patient families, and survivors. Support groups, in person or via electronic messaging platforms, were proposed interventions. All intervention themes were discussed by clinicians, caregivers, and patients across all three sites; however, the ways in which these interventions were proposed varied on the basis of cultural context (Table 3, column 3).

## DISCUSSION

Stigma is a known barrier to health-seeking behavior^2^ and has been associated with decreased care utilization,^8^ psychological distress,^13,14^ and treatment abandonment.^19,22^ Among adult patients, stigma has been described as a multilevel phenomenon that affects outcomes across the cancer continuum.^8,32-34^ We systematically applied a framework used in previous cancer stigma research^35^ to understand themes associated with stigma at multiple pediatric cancer centers in diverse LMICs. Our results demonstrate the relevance of this framework to pediatric oncology and led to an adapted stigma model (Fig 2) through which the global childhood cancer community may understand the importance of stigma. The adapted model emphasizes stigma manifestations. The concept of stigma drivers is preserved from the original framework and highlighted as the category of factors contributing to stigma. Participants in this study emphasized both negative and positive outcomes of overcoming stigma, thus these have been separated. Finally, facilitators from the initial framework have been reconceptualized into mitigators and mirrored interventions, which may form the basis of future work.

As a social phenomenon, stigma is inherently influenced by cultural context.^36^ Culture affects stigma practices, and interactions between culture and stigma influence care-seeking behavior.^37^ Nevertheless, the relationship between culture and stigma is complex. Our findings demonstrate cultural and contextual nuances as particularly related to stigma drivers. For example, in Zimbabwe and Guatemala, traditional beliefs and religion intertwine to affect cancer myths. By contrast, in Jordan, there is a pervasive faith in Allah and traditional healers are not as prevalent. Similarly, although aesthetic concerns drive stigma for both patient populations, they manifest differently for a teenage girl facing hair loss and amputation than for the parents of an infant facing enucleation. Despite these variations, the shared experience of pediatric cancer labels patients and their families as part of a stigmatized outgroup and this unifying experience affects outcomes in ways that are more alike than different. Previous research has demonstrated heterogeneity regarding stigma within a single culture^36,38^; our results suggest common experiences *within a disease* across cultures. This has broad implications as it suggests stigma might be assessed and addressed similarly for pediatric patients with cancer with different cancer types in diverse communities, a proposition that is particularly important, given pediatric cancer is a rare disease. Additionally, although this study was conducted at the time of diagnosis, participants describe anticipated stigma and reflect on outcomes that may affect pediatric patients and their families for a lifetime. We thus hypothesize that the proposed stigma model will be relevant to pediatric cancer populations around the world from diagnosis through survivorship.

Similarly, interventions identified by participants were consistent across all three sites. These interventions mirrored mitigators and could be implemented to alleviate the negative manifestations and impacts of stigma. Interventions included those with a societal focus, such as public advocacy and awareness campaigns, as well as strategies targeted at patients and families, including education and support groups. These interventions could be globally conceived and developed using standardized information. However, implementation efforts focused on community awareness campaigns, support groups, and educational materials are more likely to be impactful if they are culturally adapted and tailored for each setting and population.^39-44^ Furthermore, tools are needed to clinically assess stigma and connect patients and families at high risk with interventions. Although there are many existing tools to identify stigma in health care, none specifically address stigma as experienced by patients with pediatric cancer.

Limitations to this study include the restriction to two disease types and three centers. Further research is necessary to investigate how identified themes might manifest in other cultural contexts and pediatric cancer types. This study explored stigma around the time of diagnosis and thus does not fully capture how cancer stigma might evolve, including as it applies to childhood cancer survivors. Finally, although data were collected in three languages, analysis was conducted in English, which may have affected our capacity for nuanced interpretation.

In conclusion, stigma has been identified as a barrier to care in adult cancer populations, and a few national cancer control plans in LMICs already include stigma.^45^ Nevertheless, stigma remains understudied in pediatric cancer populations. This detailed examination of pediatric cancer stigma as perceived by clinicians, caregivers, and patients in three global settings demonstrates a shared but intersectional stigma experience that is largely retained between diverse cultures. We provide an adapted model for considering pediatric cancer stigma and its impact on outcomes including medical decision making and suggest potential interventions as the focus of future work. It is essential that pediatric cancer control efforts recognize the upfront and potential lifelong impact stigma is having on patients and families around the world and begin to implement change.
